# A Large Cross-Sectional Survey Investigating the Knowledge of Cervical Cancer Risk Aetiology and the Predictors of the Adherence to Cervical Cancer Screening Related to Mass Media Campaign

**DOI:** 10.1155/2014/304602

**Published:** 2014-06-12

**Authors:** Corrado De Vito, Claudio Angeloni, Emma De Feo, Carolina Marzuillo, Amedeo Lattanzi, Walter Ricciardi, Paolo Villari, Stefania Boccia

**Affiliations:** ^1^Department of Public Health and Infectious Diseases, Sapienza University of Rome, Piazzale Aldo Moro 5, 00185 Rome, Italy; ^2^Local Health Unit Teramo, Abruzzo Region, Circ.ne Ragusa 1, 64100 Teramo, Italy; ^3^Section of Hygiene, Institute of Public Health, Università Cattolica del Sacro Cuore, Largo Francesco Vito 1, 00168 Rome, Italy; ^4^IRCCS San Raffaele Pisana, Via della Pisana 235, 00163 Rome, Italy

## Abstract

*Objectives.* The aims of this study were to compare the characteristics of women who got a Pap-test during the mass media campaign, carried out in an Italian region by broadcasts advertising, and two years later and to identify the determinants of knowledge of cervical cancer etiology and of the adherence to the mass media campaign. *Methods.* A cross-sectional survey was carried out through a self-administered questionnaire. *Results.* A total of 8570 randomly selected women were surveyed, 823 of these had a Pap-test during the mass media campaign period and 7747 two years later. Higher educational level, being not married, and living in urban areas were the main independent characteristics associated with a higher level of knowledge of cervical cancer etiology, although a previous treatment following a Pap smear abnormality was the strongest predictor (OR = 2.88; 95% CI: 2.43–3.41). During the campaign period women had the Pap-test more frequently as a consequence of the mass media campaign (OR = 8.28; 95% CI; 5.51–12.45). *Conclusions.* Mass media campaign is a useful tool to foster cervical screening compliance; however, its short-term effect suggests repeating it regularly.

## 1. Introduction

Cancer is the third most common malignancy among females, with estimated 529,000 new cases and 275,000 deaths worldwide in 2008 [1]. According to the most recent data of the Italian Network of Cancer Registries (AIRTUM), cervical cancer accounts for 1.6% of all newly diagnosed cancers among Italian women [2]. The vast majority of cervical cancer cases are attributable to human papilloma virus (HPV), which is a sexually transmitted disease [3, 4]. The latency period between initial HPV exposure and the development of cervical cancer may be months to years. Although rapid progression is possible, average time from initial infection to manifestation of invasive cervical cancer is estimated at up to 15 years [5], thus providing a large window of action for Papanicolaou (Pap-test) screening and treatment of cervical cancer precursors.

Cervical cancer incidence and mortality rates have dropped dramatically during the 20th century in developed countries due to implementation of screening programs. According to AIRTUM data [6], the implementation of organized nationwide cervical screening (CS) programme in Italy since 1996 contributed to the decreasing mortality trends for cervical cancer in the past decade [7]. In Italy CS includes personal invitations for a Pap-test sent to women aged 25–64 years every three years by ordinary mail and a monitoring system and quality assurance of the program [8]. According to the National Centre for Screening Monitoring, 121 active programs were in place in Italy in 2007 [8], with a target population of almost 12 million women. By looking at the actual extension of CS as the ratio between the number of women regularly invited each year and the entire target group in case of full implementation, some differences emerged between Centre (above 70%), North (above 55%), and South (about 43%) of Italy, suggesting that many women are actually underscreened [9]. As regard to the screening compliance, a clear North to South gradient is observed, with the highest percentage of women who actually have the Pap-test in the northern regions (60.1%) and the lowest in the southern regions (41.7%), probably due to the more recent introduction of screening programs in southern regions (The National Centre for Screening Monitoring. Tenth report 2012. Available at: http://www.osservatorionazionalescreening.it/sites/default/files/allegati/EPv36i6s1.pdf).

Abruzzo is an Italian southern region with a population of more than 1,300 million inhabitants. Although a fully active regional CS programme has been implemented since 1999, with an actual compliance estimated around 5 67.5% (screening activity in Abruzzo region (available at: http://sanitab.regione.abruzzo.it/screening/resoconto+statistico/regione+abruzzo+al+31+dicembre+2009.pdf), to counteract the low compliance the Regional Health Authority and the CS Committee designed and conducted in 2006 a media campaign to encourage women to undergo a Pap-test. To counteract the low compliance the Regional Health Authority and the CS Committee designed and conducted in 2006 a media campaign to encourage women to undergo a Pap-test. Even though mass media campaigns have been shown to be effective in improving screening participation [10, 11], woman compliance is strongly influenced by the level of knowledge on the risk factors and the general attitude toward CS procedure [12–14]. Based on these observations, the objectives of our study were (i) to describe the characteristics of woman who got the Pap-test during the mass media campaign and two years later when the campaign was ceased; (ii) to identify the predictors of knowledge of cervical cancer aetiology; and (iii) to identify the predictors of the adherence to the mass media campaign in the Abruzzo Region.

## 2. Methods

### 2.1. Mass Media Campaign Development and Characteristics

The Abruzzo Regional Health Authority and the CS Committee designed a mass media campaign on local television stations by developing three different advertisements on: (1) Pap-test safety: it can be performed even and during pregnancy; (2) Pap-test effectiveness: it can detect early cancer signs; and (3) HPV testing: in addition to Pap-test women can be tested to detect HPV infection. The time period of the televised marketing message lasted about three months (from January to March 2006).

### 2.2. Survey Population and Data Collection

To compare the characteristics of the women who got a Pap-test during the media campaign on CS programme (Group 1) with those who got a Pap-test two years later (Group 2), we conducted a cross-sectional survey on two representative samples of women, aged from 25 to 64 years. A structured questionnaire was administered to the first sample of women through a telephone interview, while a face-to-face interview using the same questionnaire was conducted on the second sample of women. Both telephone and face-to-face interviews were carried out by the same team of specifically trained nurses, in order to minimize the risk of response bias. The source population was the entire female population of Abruzzo region aged from 25 to 64 years. Sampling for Group 1 was performed using a list-assisted method with valid phone numbers appended with random numbers using the list of women who had access to consulting rooms and other public facilities of Abruzzo region to get the Pap-test during the year 2006, and on the basis of the lists of the local health authorities of the women who got the Pap-test during the period April 2008–March 2009 for Group 2. Both samples were stratified according to the age population distribution and residence area to enrich the 15% of the potentially screenable population.

### 2.3. Survey Tool: Questionnaire Measures

A structured questionnaire was designed to collect women's sociodemographic characteristics and to assess the extent of their knowledge about CS as well as their compliance to the media campaign on CS programme. The questionnaire was focused on the women's own assessment of their knowledge of the CS and its frequency, previous Pap-test, the healthcare facility involved in Pap-test delivery, and possible surgical treatment after a positive result as well as knowledge regarding HPV infection and cervical cancer. In addition age, residence area, educational level, marital status, professional occupation, sexual activity, age at first intercourse, and contraception were collected. Finally, the reason to get the Pap-test was answered using a multiple-choice single answer question to assess if the respondents got the pap test to prevent cervical cancer, in response of the invitation letter or of the mass media campaign, after suggestion of the general practitioner/gynaecologist or after suggestion of a friend.

### 2.4. Statistical Analysis

A descriptive analysis and a univariate analysis were carried out to compare the main characteristics of the investigated groups. Two different models of stepwise logistic regression with backward elimination were performed on the overall sample of woman to identify: (i) predictors of knowledge of cervical cancer aetiology and (ii) predictors of having a Pap-test as a consequence of the mass media campaign. In the first model, women who knew that cervical cancer is caused by sexual transmission were compared with all others; in the second model, women who got the Pap-test in response to the mass media campaign were evaluated against all others. Age, residence area, educational level, marital status, previous treatment after Pap-test, and professional occupation were initially tested in all models as predictor variables. Pap-test as a consequence of the mass media campaign and knowledge about cervical cancer aetiology were also considered as main independent variables in models one and two, respectively.

Multiple logistic regression models were built as suggested by Hosmer and Lemeshow [15]. Each variable was examined by univariate analysis using the appropriate statistic test (Student *t*-test and chi-squared test) and included in the model when the *P* value was lower than 0.25. Subsequently, multivariate logistic regression with backward elimination of any variable that did not contribute to the model according to the likelihood ratio test (significance level set at *P* = 0.05) was performed. Variables whose exclusion altered the coefficient of the remaining ones were kept in the model. Interaction terms were tested using a cut-off of 0.15 significance level. Adjusted odds ratios (ORs) and 95% confidence intervals (CIs) were calculated. The statistical analyses were performed using Stata version 8.0 (College Station, Texas, Stata Corporation, 2003).

## 3. Results

A total of 8570 women were interviewed. Eight-hundred twenty-three subjects had a Pap-test during the mass media campaign period and 7747 two years later. The women who got a Pap-test during the campaign period (first group) were slightly younger than those who got a Pap-test two years after the campaign period (second group). The mean age was 42.5 years (±10.9) in the first group and 43.9 years (±10.7) in the second group (*P* < 0.001). Most of the women that got a Pap-test during the campaign period had a high school degree (45.1%) and only 8.9% had none or a primary school degree, while in the second group there were less high school degree (41.9%) and a little more women with none or primary school degree (12.7%). Almost three-fourths of the women in both groups were married, even if in the first group there were more singles (19.3% versus 16.3%) and less separated/divorced (3.9% versus 6.2%) than in the second one. The most represented occupations were housewife, employee, labourer, and teacher in both groups (Table 1).

Notably, a higher percentage of women in the first group than in the second one became aware of the screening program through the mass media campaign and got the Pap-test in response to it (11.9% versus 0.9%). Around 14.3% and 27.9% of the responders were aware that cervical cancer is attributable to sexual transmission in the first and second groups, respectively. Only 11.4% of women in the first group and 9.0% in the second group were at the first Pap-test (Table 2).

Globally, 26.6% of women knew that cervical cancer is attributable to sexual transmission. The multiple logistic regression analysis showed that knowledge about cervical cancer aetiology increases with the educational level (OR = 1.97; 95% CI: 1.83–2.12), if the women are single, widow, or divorced (OR = 1.30; 95% CI: 1.12–1.52) and if they live in urban areas in both groups (OR = 1.21; 95% CI: 1.11–1.32). A previous therapeutic treatment following a Pap smear abnormality is the strongest predictor of the adequate knowledge (OR = 2.88; 95% CI: 2.43–3.41) (Table 3).

Globally, 1.7% of woman stated that they got the Pap-test as a consequence of the mass media campaign. The results of the multiple logistic regression analysis showed that this phenomenon was higher during the mass media campaign period (OR = 8.28; 95% CI 5.51–12.45), among younger women (OR = 0.97; 95% CI: 0.95–0.99), those resident in rural areas (OR = 1.52; 95% CI: 1.06–2.18), and among those with lower knowledge of cervical cancer aetiology (OR = 0.45; 95% CI: 0.24–0.84) (Table 4).

## 4. Discussion

A meta-analysis of media health campaigns reported that campaigns promoting mammography and cervical cancer screening program reported that almost 4% of women positively change their behavior in response to a television campaign [10]. Little is currently known on the effectiveness of mass media campaign to promote Pap-test screening programs in Italy, especially in southern regions. This large cross-sectional study was specifically designed to identify the characteristics of women resident in a southern region of Italy who adhered to a local television campaign on the importance of having a Pap-test to prevent cervical cancer. The results of our study show that women adhering to the CS program as a result of the media campaign are more likely to be young, to be rural resident, and to have a poor knowledge of cervical cancer etiology. The effectiveness of the campaign to encourage women to do Pap smear analysis, however, decreased over time. These results are quite consistent with those of a recent paper [16] that reported a statistically significant 18% increase in the number of Pap-tests performed during a television campaign in Australia compared to the same period in previous years. Nevertheless, a decline to background levels was observed approximately three weeks immediately after the television campaign [16].

The results of the multivariate analysis showed that mass media campaign is a good tool to increase CS participation of women with a high risk of unscreening or underscreening due to a low level of knowledge, since poor knowledge on the risk factors for cervical cancer has been recognized as one of the most important predictors that reduces participation in CS [17]. The level of knowledge of women on cervical cancer aetiology in this study was quite low, as around 26.6% of the respondents declared to know that cervical cancer is caused by a sexually transmitted infection. A previous population-based survey of women aged 18 to 75 living in the USA found that ~40% of women had heard of HPV, but less than half of those knew that it caused cervical cancer [18]. A lower percentage of knowledge was detected among German women aged 25 to 75, since only 3.2% of them knew that infection with HPV virus is a risk factor for cervical cancer [19]. Moreover, consistently with other surveys, we found that women with adequate knowledge are more likely to have a higher level of education and to have been previously experienced with an abnormal Pap smear and positive HPV results [18].

The results of the multivariate analysis also showed that the mass media campaign was effective on rural resident women. This finding appears very important and is worthy of comment. A previous research carried out in the USA suggested that rural residents are less likely to receive timely cancer screening test, thus causing cancer diagnosis at more advanced stages [20]. This result was also reported in a case-control study carried out in Italy to evaluate the compliance to the colorectal cancer screening [21]. A possible explanation of this finding is the presence of logistical barriers and the distance from the test provider that often may limit the use of preventive care units. Subjects who live close to the provider are often more likely to comply [22], while the others perceive the prevention activity such as a screening test as a time-consuming activity.

This study has some potential limitations. Firstly, the research questions were investigated by a cross-sectional study design. As well known, such design precludes determination of causal relationships between different factors and outcomes. Secondly, information was gathered by telephone and face-to-face direct interview questionnaire, so misclassification of the covariates collected cannot be ruled out. Interviewers, however, were professionally trained nurses thus the risk of social desirability response bias should be low.

## 5. Conclusions

In conclusion, mass media campaign can enhance healthy behaviours and can track the diffusion of adequate knowledge. However, the short-term effects suggest that the media campaign should be repeated regularly to steadily increment the compliance to cervical cancer screening program.

## Figures and Tables

**Table 1:** Selected demographic and professional characteristics of the women who got the Pap-test during the campaign period (Group 1) and two years later (Group 2).

Characteristics	Group 1		Group 2		*P* value
	Mean	SD	Mean	SD	
Age, years	42.5	10.9	43.9	10.7	<0.001

	*N* = 823	%	*N* = 7747	%	

Place of residence					
Urban	450	54.68	4079	52.65	0.268
Rural	373	45.32	3668	47.35	
Educational level					
None/primary school	65	8.89	978	12.71	0.009
Middle school	223	30.51	2184	28.39	
High school	330	45.14	3223	41.90	
Academic degree	113	15.46	1307	16.99	
Missing	*55 *	*0.71 *	*92 *	*11.18 *	
Marital status					
Single	135	19.29	1239	16.30	0.025
Married	518	74.00	5662	74.49	
Separated/divorced	27	3.86	471	6.20	
Widow	20	2.86	229	3.01	
Missing	*123 *	*14.94 *	*146 *	*1.88 *	
Professional occupation					
Trader	27	3.76	299	3.90	<0.001
Laborer	101	14.05	1063	13.88	
Employee	94	13.07	1240	16.19	
Corporate entrepreneur	10	1.39	88	1.15	
Agricultural entrepreneur	8	1.11	134	1.75	
Freelancer	8	1.11	293	3.82	
Teacher	43	5.98	548	7.15	
Housewife	237	32.96	2645	34.53	
Retired	31	4.31	384	5.01	
Craftsman	1	0.14	92	1.20	
Caregiver	27	3.76	239	3.12	
Waitress	14	1.95	145	1.89	
Shop assistant	10	1.39	78	1.02	
Sanitary	16	2.23	71	0.93	
Student	9	1.25	47	0.61	
Unemployed	28	3.89	162	2.11	
Other	55	7.65	132	1.73	
Missing	*105 *	*12.76 *	*86 *	*1.11 *	

**Table 2:** Knowledge of cervical cancer aetiology and related screening procedure and motivation to get the Pap-test among women who got the Pap-test during the campaign period (Group 1) and two years later (Group 2).

Characteristics	Group 1		Group 2		*P* value
	*N* = 823	%	*N* = 7747	%	
Reason to get the Pap-test					
To prevent cervical cancer	258	35.88	2902	41.80	<0.001
Invitation letter	176	24.48	1942	27.97	
Mass media campaign	86	11.95	63	0.91	
General practitioner/gynaecologist suggestion	103	14.33	1139	16.41	
Friend suggestion	27	3.76	85	1.22	
Other	69	9.60	811	11.69	
* Missing *	*104 *	*12.63 *	*805 *	*10.39 *	
Knowledge of cervical cancer aetiology					
Yes, sexual mode	104	14.31	1994	27.87	<0.001
Yes, unknown way	321	44.15	2502	34.97	
No	302	41.54	2659	37.16	
* Missing *	*96 *	*11.66 *	*592 *	*7.64 *	
Previous Pap-test					
No	94	11.42	695	9.00	0.023
Yes	729	88.58	7023	91.00	
* Missing *	—	—	*29 *	*0.37 *	

**Table 3:** Results of the logistic regression analysis to identify predictors of knowledge of cervical cancer aetiology in the entire sample.

Variable	OR	95% CI	*P* value
Education degree* (*none/elementary school = 0; middle school = 1; high school = 2; university = 3*)	1.97	1.83–2.12	<0.001
Previous treatment after Pap-test (*no = 0; yes = 1*)	2.88	2.43–3.41	<0.001
Marital status (*married = 0; single/divorced/widow = 1*)	1.30	1.12–1.52	0.001
Place of residence (*rural = 0; urban = 1*)	1.21	1.11–1.32	<0.001

*Variable modelled as ordinal, since linearity was assessed.

**Table 4:** Results of the logistic regression model to identify the characteristics of the women who got the Pap-test as a consequence of the mass media campaign in the entire sample.

Variable	OR	95% CI	*P* value
Age (*continue*)	0.97	0.95–0.99	0.001
Place of residence (*urban = 0; rural = 1*)	1.52	1.06–2.18	0.024
Group source (0 = Pap-test after 1 year; 1 = Pap-test during the mass media campaign)	8.28	5.51–12.45	<0.001
Knowledge of cervical cancer causes (*no = 0; yes = 1*)	0.45	0.24–0.84	0.012
